# Applicability of vancomycin, meropenem, and linezolid in capillary microsamples vs. dried blood spots: A pilot study for microsampling in critically ill children

**DOI:** 10.3389/fped.2022.1055200

**Published:** 2023-01-10

**Authors:** Xu Xiaoyong, Wang Jinglin, Wang Guangfei, Zhang Huimin, Xu Hong, Li Zhiping

**Affiliations:** ^1^ Department of Clinical Pharmacy, Children’s Hospital of Fudan University, National Children’s Medical Center, Shanghai, China; ^2^ Department of Pharmacy, Union Hospital, Tongji Medical College, Huazhong University of Science and Technology, Wuhan, China; ^3^ Department of Pharmaceutics, China Pharmaceutical University, Nanjing, China; ^4^ Department of Nephrology, Children’s Hospital of Fudan University, National Children’s Medical Center, Shanghai, China

**Keywords:** capillary microsampling (CMS), DBS, antibiotics, pediatric, agreement, intensive care

## Abstract

**Introduction:**

Therapeutic drug monitoring (TDM) has been shown to be clinically beneficial for critically ill patients. However, this is a burden for neonates or children with small circulating blood volumes. Here, we aimed to develop and validate a microsampling TDM platform (including dried blood spots (DBS) and capillary microsamples (CMS)) for the simultaneous quantification of vancomycin, meropenem, and linezolid.

**Methods:**

Paired DBS and CMS samples were obtained from an intensive care unit (ICU) to evaluate its clinical application. Estimated plasma concentrations (EPC) were calculated from DBS concentrations. Agreement between methods was evaluated using Deming regression and Bland-Altman difference plots.

**Results:**

The microsampling methods validation showed excellent reliability and compatibility with the analysis of the sample matrix and hematocrit range of the studied population. The DBS and CMS accuracy and precision results were within accepted ranges and samples were stable at room temperature for at least 2 days and 8 h, respectively. Hematocrit had no impact on CMS, but sightly impacted DBS measurements. The CMS and DBS antibiotic concentrations correlated well (*r* > 0.98). The drug concentration ratio in DBS samples to that in CMS was 1.39 for vancomycin, 1.34 for meropenem, and 0.94 for linezolid. The EPC calculated from the DBS using individual hematocrit ranges presented comparable absolute values for vancomycin (slope: 1.06) and meropenem (slope: 1.04), with a mean of 98% and 99% of the measured CMS concentrations, respectively.

**Discussion:**

This study provides a microsampling TDM platform validated for clinical use for a rapid quantification of three antibiotics and is suitable for real-time TDM-guided personalization of antimicrobial treatment in critically ill children.

## Introduction

Therapeutic drug monitoring (TDM) offers a significant opportunity to improve treatment efficacy while minimizing toxicity. However, the small circulating blood volume in critically ill children does not allow for frequent determination of drug concentrations (1). Therefore, microsampling methods are urgently needed in critically ill children for TDM (2, 3). Microsampling technologies include options for wet- or dried-format sampling, such as capillary microsampling (CMS) or dried blood spot (DBS) sampling, which have implications regarding storage, transport, and determining microsample accuracy (4).

Vancomycin (VAN), meropenem (MEM), and linezolid (LZN) are frequently used in neonatal and pediatric intensive care units (ICUs) (5). However, VAN may cause severe adverse reactions, such as nephrotoxicity and ototoxicity, in children (6). The risk of nephrotoxicity increases as a function of VAN when concentration levels are above 15–20 mg/l (7). LZN demonstrates wide inter-individual variability, resulting in insufficient levels in 63% of patients suffering from treatment failure, while patients at supratherapeutic levels are susceptible to developing hematologic and neurologic toxicity (8). MEM is a time-dependent beta-lactam antibiotic with rapid elimination through the kidneys (9), and TDM is therefore recommended for patients with augmented renal clearance (10). In short, TDM can be an excellent treatment tool, along with these antibiotics, for alerting patients who may be at risk of toxicity at supratherapeutic levels or treatment failure and possible antimicrobial resistance risks at subtherapeutic levels.

Barco et al. published an analytical method for the simultaneous determination of 14 antibiotics (including VAN, MEM, and LZN) in plasma (11) but did not address microsampling. Several methods utilize DBS or CMS samples to separately determine the three antibiotics described in the literature (12–15). From a clinical standpoint, small-volume sampling and simultaneous determination of the three antibiotics could be useful in pediatric ICUs. In addition, comparative evaluation of the relationship between sampling technology and drug characteristics may be worth exploring. Here, we focus on the quantification of VAN, MEM, and LZN in the DBS and CMS samples of critically ill children.

There are two main aims of this study: the first is to set up and apply a fully validated microsampling TDM for the simultaneous determination and quantification of three antibiotics in DBS and CMS samples; the second is to evaluate the reliability of two microsampling methods applied in routine TDM in critically ill children.

## Materials and methods

### Reagents and chemicals

The reference standard of vancomycin (Lot No.130360-202103), meropenem (Lot No.130506), and linezolid (Lot No.130640-201901) complied with ChP Reference Standards. The internal standards (IS) for norvancomycin (Lot No.130338-201704), meropenem-2H6 (Lot No.21B063-A5), and linezolid-2H3 (Lot No.21B035-A1) were purchased from ZZBIO (Shanghai, China). Ultrapure water was provided by a smart2Pure pro water purification system (Thermo Scientific, USA). Acetonitrile (ACN), methanol (MeOH), and formic acid (FA) were obtained from Sigma-Aldrich (Shanghai, China), and all solvents were of LC-MS grade. Whatman 903® paper was purchased from GE Healthcare, and capillary tubes (70 µl) were purchased from Hirschmann Co. Ltd (Eberstadt, Germany).

Stock solutions of VAN, MEM, and LZN were prepared in MeOH at concentrations of 4, 4, and 2 mg/ml, respectively. All stock solutions were stored at −80°C and were further diluted in 50% MeOH to obtain a working solution. All individual IS stock solutions were prepared in ACN at 1 mg/ml and also stored at −80°C. The IS working solution consisted of 2.5 µg/ml norvancomycin, 5.0 µg/ml meropenem-2H6, and 1.0 µg/ml linezolid-2H3.

### Calibrators and quality control (QC) samples

Calibrators were prepared at eight concentrations, based on therapeutic ranges, by spiking working solutions to blank human plasma and whole blood at concentrations of 1, 2, 4, 8, 15, 30, 50, and 100 µg/ml for VAN; 0.4, 0.8, 1.6, 3.2, 6, 12, 20, and 40 µg/ml for MEM; and 0.2, 0.4, 0.8, 1.6, 3, 6, 10, and 20 µg/ml for LZN. QC samples [lower limit of quantification (LLOQ), low, medium, and high, respectively] were prepared by spiking working solutions to blank plasma and whole blood at 1, 3, 7.5, and 75 µg/ml for VAN; 0.4, 1.2, 3, and 30 µg/ml for MEM; 0.2, 0.6, 1.5, and 15 µg/ml for LZN. The amount of solvent added to the matrix, for both the calibrators and QCs, did not exceed 5%. Calibrators and QC samples were aliquoted and stored at −80°C until analysis.

### Sample preparation

All samples were subjected to protein precipitation. To prepare the CMS samples, the whole blood samples of patients were filled in capillary tubes and sealed with wax on both ends. The CMS samples were sent to the lab and centrifuged for 5 min at 3,000 rpm to separate the plasma and blood cells. The volume of the plasma sample was estimated by the length of the plasma fraction and cut down by special capillary pliers. The cut plasma fraction was washed using blank plasma (for standardizing the plasma volume to 50 µl), 15 µl formic acid, and 50 µl ACN in turn. Similarly, the STD and QC samples were prepared using an aliquot of 50 µl calibrator and QC plasma samples, mixed with the same reagents. All samples were mixed with 150 µl IS working solution, shaken at 2,000 rpm for 5 min and centrifuged for 5 min at 12,500 rpm. 10 µl supernatant was transferred and diluted 20-fold in water. Finally, 5 µl diluent was injected into the LC-MS/MS for analysis.

For DBS samples, an aliquot of 50 µl whole blood samples was used for the calibration curve. QC and unknown samples were spotted on Whatman 903® paper cards and dried in a fume hood at an ambient temperature for at least 30 min. DBS samples were cut off at 10 mm diameter and transferred into Eppendorf tubes. Subsequently, 200 µl of aqueous solution (containing 2% FA, v/v) and 200 µl of IS working solution was added into the tubes. Samples were shaken at 2,000 rpm for 20 min at room temperature and 5 min at 12,500 rpm. The supernatant was diluted and injected in the same manner as the CMS samples.

### Chromatographic analysis

Chromatographic analysis was performed using an ACQUITY UPLC system (Waters Corporation, USA). Mobile phases consisting of 0.2% FA in water (A) and ACN (B) at a flow rate of 0.3 ml/min were used. Separation of the components was achieved by gradient elution: the mobile phase B gradient was initiated at 10% B at 0.5 min then linearly increased to 70% at 2.8 min and to 90% at 3 min. This was maintained at 90% at 3.3 min, continuously decreased to 10% at 3.5 min, and then maintained at 10% until the end of the run, resulting in a total run time of 4 min. The UPLC system was equipped with an Acquity UPLC® HSS T3 column (100 mm length × 2.1 mm i.d., 1.8 µm) (Waters Corporation, USA), fitted with a corresponding BEH C18 guard column, and maintained at 40°C. The autosampler temperature was set at 10°C.

The LC system was coupled to a Xevo TQ-S Micro triple quadrupole mass spectrometer (Waters Corporation, United States), equipped with an electrospray ionization (ESI) source operating in positive ion mode. Multiple reaction monitoring (MRM) was applied for the detection of the components. The desolvation temperature was 550°C, the cone gas flow was 150 L/hr, and the desolvation gas flow was 900 L/hr. MRM transitions with corresponding MS parameters for quantifiers, qualifiers, and ISs are listed in Table 1. All UPLC–MS/MS data were collected and processed by Masslynx 4.1 software (Waters Corporation, USA).

**Table 1:** Retention time, MRM transitions and compound-specific MS settings.

Compound	Retention time (min)	Parent ion (m/z)	Daughter ion (m/z)	Cone (V)	Collision
Vancomycin	1.88	725.9	144.2	40	15
Meropenem	2.03	384.02	141.2	25	12
Linezolid	2.75	338.03	296.02	12	18
Norvancomycin	1.82	718	144.2	40	13
Meropenem-2H6	2.03	390.2	146.8	12	18
Linezolid-2H3	2.75	341.1	297	12	18

### Method validation

The method validation followed the Chinese Pharmacopeia guidance and bioanalytical method validation from ICH. Specifically, selectivity was evaluated by endogenous interference peaks at the retention time of VAN, MEM, LZN, and IS of blank samples from six individual whole blood samples. Linearity was validated by calibration curves. Peak area ratios to IS were taken as the ordinate, and the nominated concentrations of the three antibiotics were taken as the abscissa and fitted to a 1/x2 weighted linear regression. For the intra- and inter-batch precision and accuracy, six replicates of QC and LLOQ samples were determined at three analytical batches. Accuracy was accepted within the range of 85%–115% while the precision coefficient of variance (CV) was below 15%. The matrix effects were carried out at low, medium, and high concentrations. Blank plasma and DBS samples from six different individuals were prepared, and a working solution was then added to the extractant in order to evaluate the matrix effects. The stability of plasma and DBS samples in different storage conditions was evaluated. Samples were stored at room temperature, 2–8°C, 35°C, and −80°C, and the post-treated stability of the autosampler was evaluated by examining two levels of QC samples (low and high). Samples were considered stable if concentrations were within the range of 85%–115%. The dilution integrity of the plasma samples was processed. Extra-high QC plasma samples at a nominal concentration of 125 µg/ml for VAN, 50 µg/ml for MEM, and 25 µg/ml for LZN (1.25 times the ULOQ concentration) were diluted 10:1 with blank plasma. Six replicates of the diluted samples were processed and analyzed.

For the CMS accuracy and adsorption test, six replicates of QC samples were determined. The impact of the hematocrit (Hct) on antibiotic DBS measurements was also tested. Blood samples with Hct levels of 15, 20, 25, 30, 35, and 40% were obtained from whole blood, and plasma was then added or removed. QCL and QCH DBS samples were prepared from the blood and analyzed in triplicate at a fixed Hct level of 30%. The influence of the Hct percentage on VAN, MEM, and LZN determinations was expressed as the percentages of nominal concentrations measured in DBS samples. Acceptance criteria were values between 85% and 115%.

### DBS sampling and CMS method comparison

From the drug concentration in DBS samples and the Hct, an estimated plasma concentration (EPC) was calculated using the formula:

$$\mathrm{EPC}=\frac{C_{DBS}}{1\;-\;\left(\mathrm{Hct}/100\right)}.$$

EPC was evaluated using both the individual and average Hct of the group of patients. Agreement between methods was evaluated using Deming regression and the Bland–Altman difference plot. Statistical analyses were performed with R4.04 software.

### Clinical applications

A large whole blood sample set was drawn, for routine TDM purposes, from pediatric patients receiving VAN, MEM, and LZN for the treatment of various infections at the ICU of the Children's Hospital of Fudan University, Shanghai, China. The patients were not selected in advance, and samples were included as they were received in our laboratory for routine TDM. DBS and CMS samples were produced from these whole blood samples, which were maintained at –−80°C until analysis. This study was in compliance with the Declaration of Helsinki and was approved by the local ethics committee at the hospital.

## Results

### Method validation of capillary plasma and DBS

**Figure 1:**
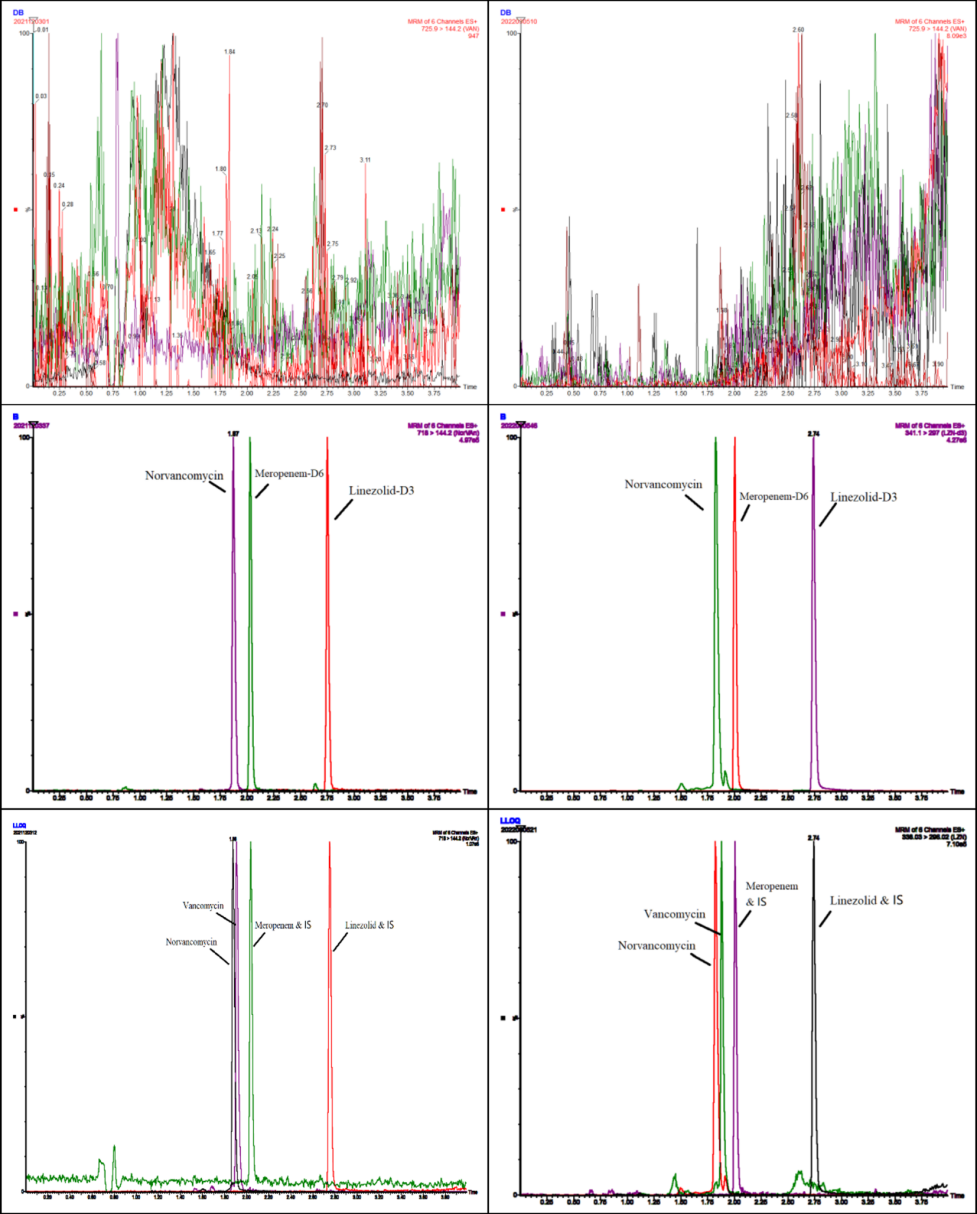
Representative chromatograms of blank, internal standard, and lower limit of quantification (LLOQ) of vancomycin, meropenem, and linezolid obtained from plasma (left) and DBSs (right).

Typical chromatograms obtained from plasma and DBS samples of VAN, MEM, and LZN in double blank, blank with internal standards, and LLOQ are illustrated in Figure 1.

There was no endogenous substance interference between analytes and the internal standard. Linearity was acceptable using a 1/x2 weighting over a range of 1–100, 0.4–40, and 0.2–20 µg/ml for VAN, MEM, and LZN, respectively. The results of the two matrixes (plasma and DBS) for the intra- and inter-assay precision and accuracy tests were all within the acceptable ranges (Table 2). The matrix effects for three antibiotics at low, medium, and high concentrations fully complied with the testing criteria (Table 3). The precision (%CV) of dilution integrity was <15% and the accuracy was within 85%–115% for all analytes. The %CV was 4.7%, 2.3%, and 2.0% for VAN, MEM, and LZN, respectively, suggesting that the plasma samples can withstand ten-fold dilution without compromising sample integrity.

**Table 2:** Results of accuracy and precision for vancomycin, meropenem, and linezolid in plasma, DBS, and capillary microsamples.

			Within-run (*n* = 6)		Between-run (*n* = 6)			Within-run (*n* = 6)		Between-run (*n* = 6)			Within-run (*n* = 6)		Between-run (*n* = 6)	
Matrix	QC level	Nominal concentration (µg /ml)	CV%	Bias%	CV%	Bias%	Nominal concentration (µg /ml)	CV%	Bias%	CV%	Bias%	Nominal concentration (µg /ml)	CV%	Bias%	CV%	Bias%
Drug		Vancomycin					Meropenem					Linezolid				
Plasma	LLOQ	1.00	3.7	−9.8	4.0	−6.4	0.400	5.6	−4.0	4.5	−2.2	0.200	3.7	−0.4	3.3	2.3
	Low	3.00	4.0	12.7	4.0	12.3	1.20	2.8	−2.6	4.3	−4.8	0.600	1.2	5.4	2.7	6.5
	Medium	7.50	1.8	6.7	3.7	9.0	3.00	1.3	0.4	3.9	−2.5	1.50	1.1	3.4	1.5	2.9
	High	75.0	1.4	−5.7	4.0	−1.7	30.0	1.3	−5.8	3.1	−6.7	15.0	1.9	−5.9	2.4	−6.8
DBS	LLOQ	1.00	4.5	6.1	3.7	6.2	0.400	4.7	9.1	9.8	8.0	0.200	2.3	4.4	2.7	4.0
	Low	3.00	2.3	1.6	3.2	1.9	1.20	2.3	7.3	5.2	4.7	0.600	1.1	5.0	1.4	4.8
	Medium	7.50	1.5	−2.8	3.4	−5.6	3.00	1.1	−2.6	7.3	−9.1	1.50	0.7	−6.6	2.6	−8.8
	High	75.0	2.8	−0.5	3.3	−0.1	30.0	2.1	5.5	3.8	2.1	15.0	2.9	1.1	2.7	−0.7

**Table 3:** Results of the matrix effect for vancomycin, meropenem, and linezolid in plasma and DBS (*n* = 6).

Matrix	QC level	Vancomycin		Meropenem		Linezolid	
		mean%	SD%	mean%	SD%	mean%	SD%
Plasma	Low	98.3	2.1	109.0	3.1	118.5	2.9
	Medium	98.9	0.7	108.3	3.2	117.3	1.8
	High	100.3	1.5	111.9	2.7	109.4	2.0
DBS	Low	98.7	3.0	91.1	3.0	95.6	1.5
	Medium	94.8	3.5	94.1	6.0	90.8	2.1
	High	99.0	2.6	94.2	5.7	94.8	2.8

The results of the stability testing of VAN, MEM, and LZN in plasma are shown in Table 4. VAN and LZN in plasma were stable at 2–8°C for 24 h, at −80°C for at least 179 days, and at room temperature for 1 day. However, MEM in plasma was stable at 2–8°C for 24 h, at −80°C for 70 days. and at room temperature for 8 h. Three consecutive freeze-thaw cycles for all three antibiotics did not show any notable degradation. DBS cards, dried and stored in sealed bags, showed that VAN and LZN can be considered stable for at least 3 days in DBS form at both concentration levels at 4–8°C, 35°C, and room temperature and at −80°C for 73 days. In contrast, MEM showed a marked decay at a storage temperature of 35°C in DBS form for 2 days and less than a 15% loss over 30 h of storage time. During storage at 4–8°C and room temperature, MEM was stable in stabilized plasma for 2 days. Similarly, MEM stored at −80°C was stable for 73 days in DBS form.

**Table 4:** Results of stability for vancomycin, meropenem, and linezolid in plasma and DBS (*n* = 3).

DBS									
Drug	Vancomycin			Meropenem			Linezolid		
Condition	4°C	RT	35°C	4°C	RT	35°C	4°C	RT	35°C
Period	3 days	3 days	3 days	2 days	2 days	1 day	3 days	3 days	3 days
QCL	6.6	−5.0	−12.2	2.9	−0.7	−7.4	−7.4	−6.4	−10.1
QCH	−2.3	0.5	2.1	−2.5	−8.9	−9.9	−1.7	−0.5	−6.3
Plasma									
Drug	Vancomycin			Meropenem			Linezolid		
Condition	4°C	RT	−80°C	4°C	RT	−80°C	4°C	RT	−80°C
Period	1 day	1 day	214 days	1 day	8 h	70 days	1 day	1 day	199 days
QCL	6.0	−10.2	−11.1	−8.2	0.5	−7.5	−4.3	−11.8	−2.6
QCH	−5.7	1.5	10.6	−4.6	−10.2	−6.9	−5.5	0.0	2.8

**Figure 2:**
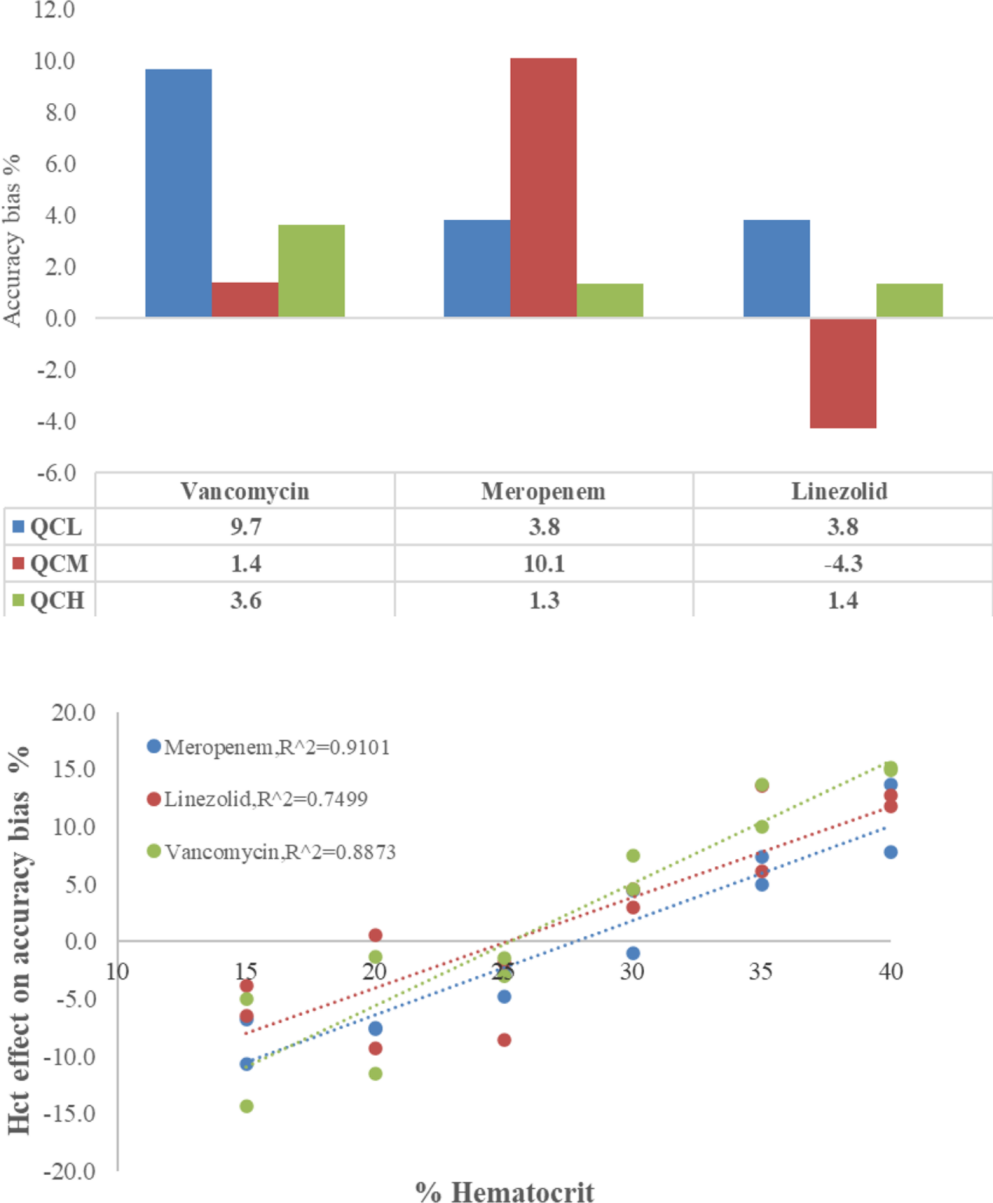
Accuracy of vancomycin, meropenem, and linezolid in capillary microsamples and DBS samples — upper: adsorption test on the capillary tube, lower: the Hct effect on DBSs.

The capillary tube absorption test and the Hct effect on DBS samples are shown in Figure 2, revealing the accuracy of determinations of VAN, MEM, and LZN. The accuracy of CMS showed an insignificant trend in the range of 95.7–110.1%, demonstrating that the three antibiotics have unspecific surface adsorption to the capillary tube. Therefore, the capillary tube could be deemed a plasma separator tube in this study. Conversely, the accuracy bias trend in DBS samples caused by sample Hct was obvious and was reportedly caused by blood viscosity. Although accuracy was systemically biased, the results remained within the Hct acceptance range of 15%–40%, with a bias of −5.0%–14.9% for VAN, −10.7%–13.6% for MEM, and −3.8%–12.7% for LZN. Therefore, results obtained with calibration curves prepared with whole blood at Hct levels of 30% are suitable for quantifying antibiotics in DBS samples from patients with Hct levels between 15% and 40% with acceptable systematic bias.

### Clinical application and method comparison

**Figure 3:**
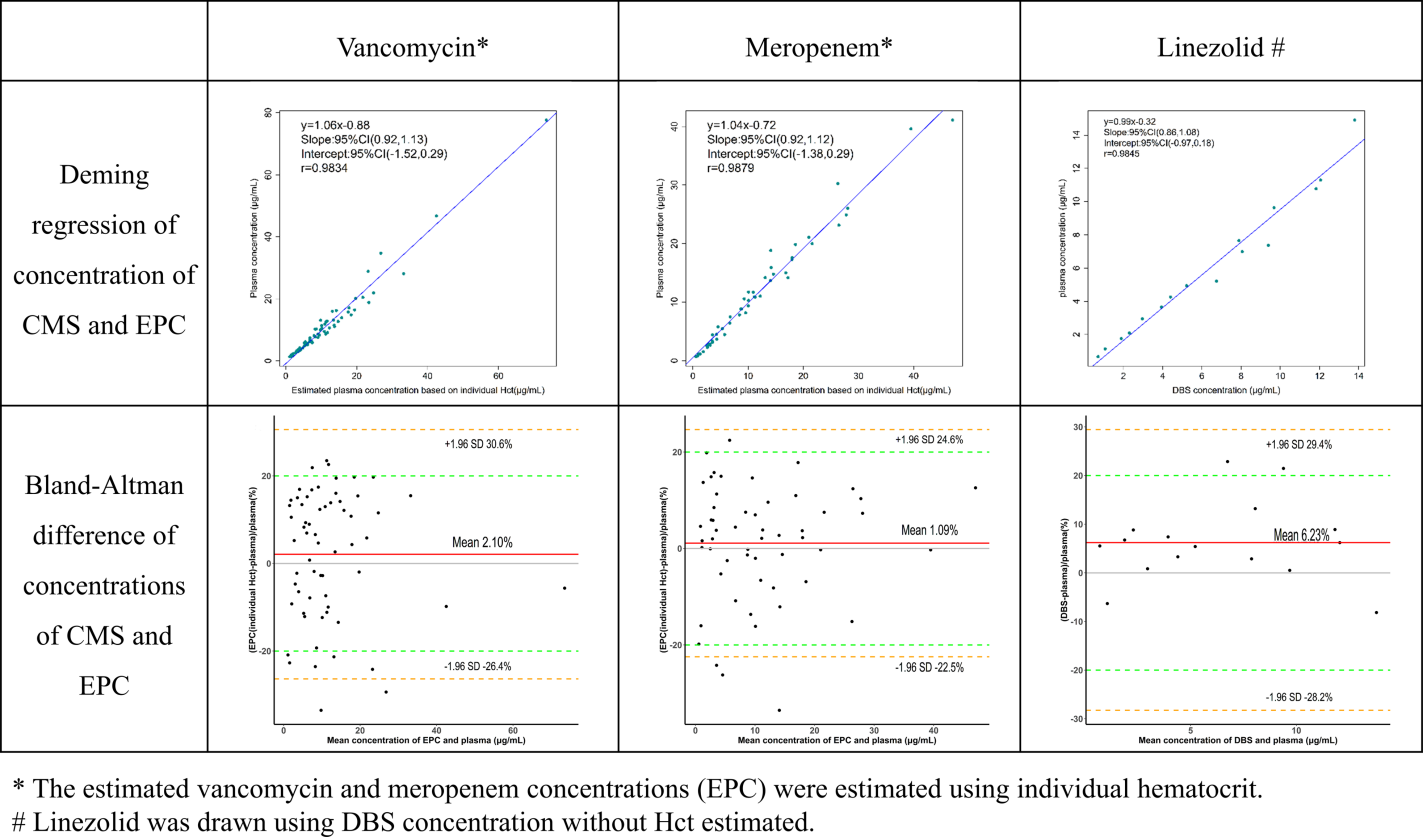
Agreement of three antibiotic concentrations between capillary microsamples and DBS samples.

After validating the pretreatment and analysis workflow of samples, the microsampling method was successfully applied to a total of 130 paired CMS and DBS samples from 97 pediatric patients, obtained from neonatal or pediatric ICUs. The measured and calculated concentrations are presented in Table 5. Three DBS and CMS antibiotic concentrations were significantly correlated (*r* > 0.98, *p* < 0.01). However, CMS to DBS drug concentration ratios were highly variable. VAN ranged from 1.03 to 1.95 with an average of 1.39, MEM from 1.04 to 1.93 with an average of 1.34, and LZN from 0.77 to 1.08 with an average of 0.94, indicating that LZN could be accurately determined in DBS cards (Deming regression: *y* = 0.99 *x* −0.32) (Figure 3). However, VAN and MEM concentrations in DBS samples showed a marked bias compared to those in CMS samples. We suspect that red blood cells may act as a diluent to the VAN and MEM plasma; therefore, it is necessary to evaluate the EPC from the DBS measurements.

**Table 5:** Characteristics of paired microsamples.

Characteristics	Vancomycin	Meropenem	Linezolid
Age, years, median (min/max)	4.9(0.05/15)	4.2(0.07/13)	1.8(0.17/8)
Source of samples, N, (PICU/NICU)	46/16	47/6	11/5
Hematocrit %, mean (min/max)	29.5(17.7/38.8)	25.8(17.3/34.5)	26.1(20.4/39.0)
Plasma concentration, µg/ml, mean (min/max)	11.86(1.35/77.73)	10.77(0.68/41.11)	5.59(0.67/14.91)
DBS concentration, µg/ml, mean (min/max)	8.51(1.02/52.69)	8.15(0.41/34.99)	6.37(0.71/13.79)
Plasma/DBS ratio, mean (min/max)	1.39(1.03/1.95)	1.34(1.04/1.93)	0.94(0.77/1.08)
Plasma/individual EPC ratio, mean (min/max)	0.98(0.77/1.34)	0.99(0.78/1.34)	/
Plasma/average EPC ratio, mean (min/max)	0.98(0.72/1.37)	0.99(0.77/1.43)	/
Percentage of paired samples with positive agreement^a^	83.8% (52/62)	92.5% (49/53)	87.5% (14/16)

^a^ The agreement acceptance criteria for paired samples were ± 20%.

Critically ill children commonly experience anemia and hemodilution due to medical interventions. The average Hct in these children (17%–39%) was markedly lower than that of normal children (35%–45%). Deming regression and Bland-Altman difference in concentrations of VAN and MEM obtained in CMS samples and EPCs were compared in Figure 3. Whether the EPC was estimated using individual or average Hct, VAN and MEM exhibit a high degree of agreement (*r* > 0.98, *p* < 0.01). The slope value from the Deming regression was 1.04 vs. 1.06 (*p* < 0.01) for VAN and 1.04 vs. 1.04 (*p* < 0.01) for MEM. However, the EPC estimated using individual Hct performs better at fitting than the EPC estimated using average Hct by the Bland-Altman difference plots. As shown in the diagram, 83.8% (52/62) of the VAN EPC estimated using individual Hct did not differ by more than 20% from the CMS samples when compared to 77.4% (48/62) using average Hct. In addition, the MEM EPC showed the same trend (92.5% (49/53) vs. 84.9% (45/53).

## Discussion

Here, we developed a microsampling TDM platform for the simultaneous quantification of empirical antibiotics commonly prescribed in critically ill pediatric patients. In comparison with outpatients, critically ill children in an ICU could have a venous catheter, which is suitable for venous microsampling. A total of 72.9% (43/59) of pediatric patients in our study received MEM treatment in combination with VAN or LZN. Microsampling and combined detection appear to reduce the blood volumes drawn from patients and provide plasma concentrations of multiple drugs.

We showed that the VAN and MEM concentration differences in DBS and CMS samples can be explained by the formula based on Hct. However, LZN showed a positive agreement between DBS and CMS samples, a result similar to that found in a previous study (16). This resulted in a blood cell-to-plasma partitioning being 0.139 ± 0.036 for VAN, 0.115 ± 0.031 for MEM, and 0.855 ± 0.080 for LZN (*n* = 9), where the LZN was homogeneously distributed in the blood. Alternatively, the CMS samples were not affected by Hct and were considered the closest microsamples to the “gold standard” of plasma sampling (4). Different sample pretreatment methods were developed to solve the problem of adsorption before validation. We compared the desorption solvent with pure water, acetonitrile, formic acid, and plasma. The results showed that blank plasma could extract antibiotics from the capillary tube and replenish plasma volumes to the standard sample preparation.

Critically ill children often have low hematocrit values. Red blood cells seem to play a dilution role for some hydrophilic or macromolecule drugs in DBS measurements (17). However, when this effect was considered and an EPC was calculated, individual Hct values performed better than average Hct values.

In clinical practice, considering the costs of equipment and training, DBS sampling and CMS seem superior to specialized sampling technologies (18) such as volumetric absorptive microsampling and solid-phase micro-extraction. DBS sampling and CMS would be acceptable to phlebotomists who can easily obtain high-quality samples. However, each technique has its own advantages and disadvantages—DBS sampling performs better in stable conditions, is robust with regards to modes of transport, and is suitable for remote community hospitals and home sampling, but the effects of Hct may have an impact on DBS samples. Therefore, DBS sampling could be a useful tool for unstable or hydrophobic (evenly distributed in the blood) drugs such as MEN, LZN, and immunosuppressants (19–21). CMS is commonly used in children's hospitals and shows a resistance to the effects of Hct; however, analytes may be surface-adsorbed in capillary tubes. Drugs with low adsorption and good stability, such as VAN, are suitable for CMS. Finally, the applicability and evaluation of microsampling methods in TDM platforms need further research.

The primary limitation to the generalization of these results is the direct comparison of DBS sampling and CMS results without comparing the results of each microsampling procedure with those of a procedure using traditional large-volume sampling. Although CMS of plasma could be accurate and precise using a full method validation (accuracy and diluted effect test) (22, 23), it should not be ruled out as potentially biased toward sources of analytical error.

## Conclusions

Here, we developed and validated a microsampling TDM platform for the accurate quantification of VAN, MEM, and LZN. CMS and DBS sampling can be used for TDM and the personalization of antibiotic therapy in critically ill children or even in neonates.

## Data Availability

The original contributions presented in the study are included in the article/Supplementary Material, further inquiries can be directed to the corresponding author/s.
