# Supplementary material for: Applicability of vancomycin, meropenem, and linezolid in capillary microsamples vs. dried blood spots: A pilot study for microsampling in critically ill children
Source: Front Pediatr. 2023 Jan 10;10:1055200. doi: 10.3389/fped.2022.1055200 (PMC9872121; doi:10.3389/fped.2022.1055200)
Supplement: Supplementary file 1 [file Datasheet1.pdf]

<table>
<tr><th></th><th>Vancomycin</th><th>Meropenem</th></tr>
<tr><td>Distribution of CMS and DBS concentration</td><td>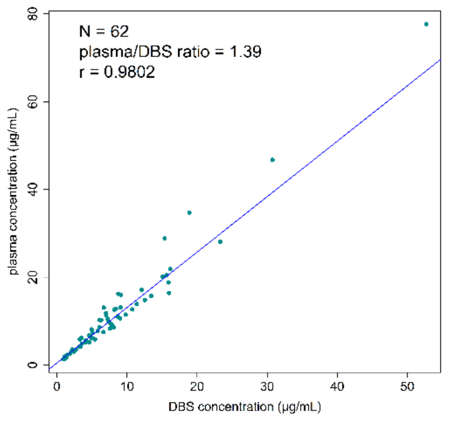
</td><td>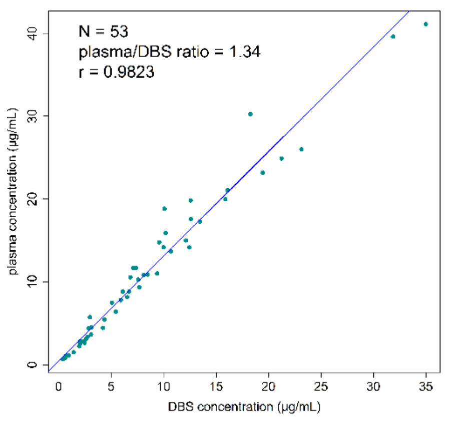
</td></tr>
<tr><td>Deming regression of concentration of CMS and EPC *</td><td>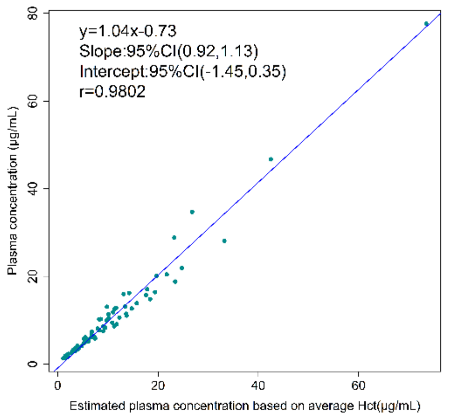
</td><td>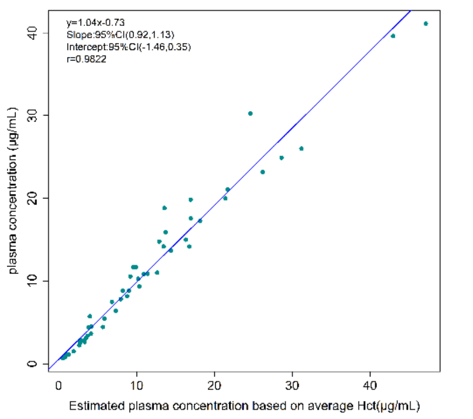
</td></tr>
<tr><td>Bland-Altman difference of concentrations of CMS and EPC *</td><td>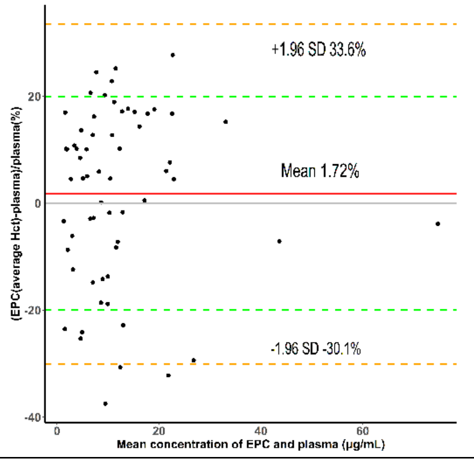
</td><td>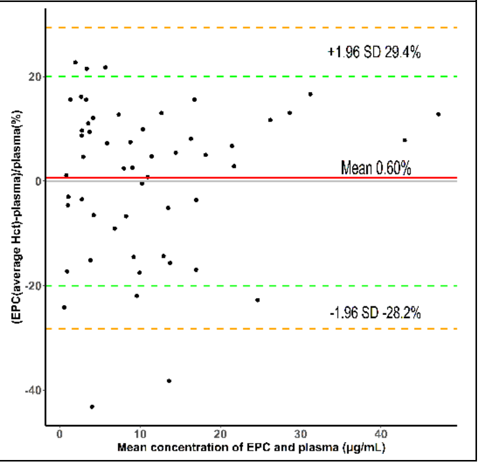
</td></tr>
</table>

* The estimated vancomycin and meropenem concentrations (EPC) were estimated using average Hct.

Supplementary Figure. 1. Distribution of vancomycin and meropenem concentration in capillary microsamples and DBS.
